# Group penalized generalized estimating equation for correlated event-related potentials and biomarker selection

**DOI:** 10.1186/s12874-020-01103-x

**Published:** 2020-08-31

**Authors:** Ye Lin, Jianhui Zhou, Swapna Kumar, Wanze Xie, Sarah K. G. Jensen, Rashidul Haque, Charles A. Nelson, William A. Petri Jr, Jennie Z. Ma

**Affiliations:** 1 University of Virginia, Charlottesville, US; 2 Harvard University, Cambridge, US; 3 Boston Children’s Hospital, Boston, US; 4 International Centre for Diarrhoeal Disease Research, Dhaka, Bangladesh

**Keywords:** Event-related potentials, Correlated data, Penalized generalized estimating equations (GEE), Variable selection, Structured correlation matrix

## Abstract

**Background:**

Event-related potentials (ERP) data are widely used in brain studies that measure brain responses to specific stimuli using electroencephalogram (EEG) with multiple electrodes. Previous ERP data analyses haven’t accounted for the structured correlation among observations in ERP data from multiple electrodes, and therefore ignored the electrode-specific information and variation among the electrodes on the scalp. Our objective was to evaluate the impact of early adversity on brain connectivity by identifying risk factors and early-stage biomarkers associated with the ERP responses while properly accounting for structured correlation.

**Methods:**

In this study, we extend a penalized generalized estimating equation (PGEE) method to accommodate structured correlation of ERPs that accounts for electrode-specific data and to enable group selection, such that grouped covariates can be evaluated together for their association with brain development in a birth cohort of urban-dwelling Bangladeshi children. The primary ERP responses of interest in our study are N290 amplitude and the difference in N290 amplitude.

**Results:**

The selected early-stage biomarkers associated with the N290 responses are representatives of enteric inflammation (days of diarrhea, MIP1b, retinol binding protein (RBP), Zinc, myeloperoxidase (MPO), calprotectin, and neopterin), systemic inflammation (IL-5, IL-10, ferritin, C Reactive Protein (CRP)), socioeconomic status (household expenditure), maternal health (mother height) and sanitation (water treatment).

**Conclusions:**

Our proposed group penalized GEE estimator with structured correlation matrix can properly model the complex ERP data and simultaneously identify informative biomarkers associated with such brain connectivity. The selected early-stage biomarkers offer a potential explanation for the adversity of neurocognitive development in low-income countries and facilitate early identification of infants at risk, as well as potential pathways for intervention.

**Trial registration:**

The related clinical study was retrospectively registered with https://doi.org/ClinicalTrials.gov, identifier NCT01375647, on June 3, 2011.

## Background

Event-related potentials (ERPs) have been widely used in studies of perceptual and cognitive development. ERPs represent the volume-conducted electrical signals generated by large populations of synchronously activated neurons activated in response to stimuli. Specifically, with multiple electrodes on the scalp, ERPs are small parts of electroencephalogram (EEG) recording of the brain response elicited to specific stimuli such as viewing pictures or words on the computer screen [1]. As the brain response to a single stimulus is usually weak or noisy in the EEG recording of a single trial, an ERP waveform is actually generated from the aggregated EEG recordings over many trials for better brain response measuring [2, 3]. In general, an ERP waveform consists of a series of positive and negative voltage deflections, characterized by the amplitudes of negative- or positive-going peaks or the latencies to these peaks in milliseconds (ms). For example, the N290 component surfaces as a negative deflection in voltage and with a peak latency between 250 and 350 ms, while the P400 component appears as a positive-going waveform that peaks between 350 and 450 ms depending on the age of the child [4–6]. Consequently, ERP data (amplitudes or latencies) are hierarchical in that there are multiple ERP measurements for each subject corresponding to multiple treatment or stimulus conditions and multiple channels (i.e., electrodes), while channels are further clustered in different regions of the brain. Comparisons of brain activities between different treatment conditions for different channels in different brain regions are of research interest [7].

In the previous literature, there are a few approaches to compare ERPs between different stimulus conditions from multiple channels. One approach is to compare ERPs between conditions for each channel individually, which is often subjected to multiple comparison problem. Lage -Castellanos et al. [8] applied false discovery rate method and performed a permutation test for comparisons within each channel and at each time point. Causeur et al. [9] introduced a dynamic factor model for multiple testing to account for the dependence among hypotheses. The second approach is to analyze the data from all channels simultaneously. One popular tactic is to group the channels by the brain regions such as frontal, central and parietal, and then perform Analysis of Variance (ANOVA) separately for each region, or include region as a factor in Multivariate Analysis of Variance (MANOVA) for all ERPs together [10]. Yet another approach is to average the ERPs over the multiple channels of interest and then compare conditions using one-way ANOVA. Either way, the channels within a brain region would be treated the same and the variations or the correlation structure between individual channels would not be accounted for. In fact, ERP measures do not only vary but also are highly correlated among channels. Vossen et al. [11] showed the correlated structure among ERP data and applied mixed regression approach. However, they only considered the correlation among repeated measurements from different conditions while channels are still modeled separately. To improve estimation efficiency, a model accounting for both the individual channel effects and the correlation structure is highly desired in ERP data analysis.

In addition to evaluating the effect of treatment conditions on the brain response of interest in ERP data, motivated by our clinical study, we are also interested in that whether such brain response is attributable to a set of important clinical risk factors and biomarkers. Since a large number of risk factors and biomarkers are available in the clinical study, variable selection using penalized methods would be preferred for such high-dimensional data to select the important predictors and estimate their impacts on the brain response. Many penalized methods have been developed based on different penalties for high-dimensional data, such as Least Absolute Shrinkage and Selection Operator (LASSO) [12], Smoothly Clipped Absolute Deviation (SCAD) [13], Elastic Net [14] and Adaptive LASSO [15]. Penalized methods for correlated data have also been proposed for marginal models [16] and for mixed effects models [17]. In addition, Wang et al. [18] proposed penalized generalized estimating equations (PGEE) for high-dimensional correlated data based on SCAD penalty. However, these available methods are not readily applicable to ERP data mainly due to the lack of consideration of the specific structured correlation among different channels in ERP data, especially when both conditions and channels are included. Second, the SCAD-based PGEE does not allow group variable selection, which is pivotal in the clinical studies as many risk factors or biomarkers are clustered or potentially correlated.

In this paper, we extend the PGEE method to a Group Penalized Generalized Estimating Equations (GPGEE) that can accommodate a multi-level structured correlation and achieve group-wise variable selection. Thus our proposed method can be readily applied to test the condition difference in ERP measures and simultaneously perform group variable selection to identify important predictors associated with ERP for brain response. To our knowledge, hierarchical models with complex correlation structure are rarely used for ERP response analysis in the ERP research, nor are the regularized regression methods with penalty. Our modeling development was motivated by the ERP data from a birth cohort of Bangladeshi children, the Performance of Rotavirus and Oral Polio Vaccines in Developing Countries (PROVIDE) study. A large and comprehensive set of non-invasive biomarkers were developed in the PROVIDE study from fecal and blood samples [19]. Children in low-resource communities such as those in the PROVIDE cohort are exposed to numerous adversities, including malnutrition, infectious disease exposure, and extreme poverty. In turn, exposure to early adversity can limit their cognitive developmental potentials with long lasting effects. Using EEG as a neuro-imaging tool for cognitive and neural development assessment, a subset of children in the PROVIDE birth cohort were measured at 3 years of age for ERP response. The primary objective of our clinical ERP study was to evaluate the impact of early adversity on brain connectivity and identify risk factors and biomarkers associated with the brain response. With the challenges and limitations in ERP research described earlier, the GPGEE model is developed to achieve the clinical objective.

Our method addresses the following major challenges in analyzing the ERP data from the PROVIDE study. First, due to the design of experiment, ERP data are hierarchical or multilevel by nature with multiple conditions and multiple channels for each study subject. Second, ERP data are highly correlated across channels under each condition, and across conditions for each channel. Lots of information would be lost by simply averaging ERPs over these channels to compare ERPs between conditions. Third, although variable selection methods for high-dimensional data have been intensively studied [12, 13] and applied in clinical and genetic studies [19–21], to our knowledge, no variable selection technique has been applied to ERP data. Further, since many predictors in the high dimensional data are categorized with multiple levels or potentially correlated, group penalty needs to be imposed in the variable selection process to ensure informative predictors and groups can be correctly selected.

The rest of the paper is organized as follows. In “Methods” section, we present the models for high-dimensional correlated data, propose the model specifically for the structured correlation matrix in ERP data, expand the PGEE method to allow group penalty, and derive the algorithm for solving group-penalized estimating equations. In “Simulations” section, we conduct a simulation study to compare the relative performance of our proposed GPGEE with the existing model under several scenarios, without and with group penalty, and with different correlation structures. In “Results” section, we apply our proposed method to ERP data from the PROVIDE study. Compared to the existing methods such as regularized regression or PGEE, our proposed method doesn’t only model the ERP multi-level structure appropriately, but also promotes group-wise variable selection. The simulation results show that our proposed method outperforms the existing modeling approaches in variable selection and parameter estimation. Our work would be one of the pioneering efforts in ERP research to test the difference in ERPs between conditions while identifying important biomarkers associated with ERPs simultaneously.

## Methods

### Clinical data and ERP measurements

The PROVIDE (Performance of Rotavirus and Oral Polio Vaccines in Developing Countries) study was a randomized controlled clinical trial with a 2-by-2 factorial design to investigate the efficacy of Rotavirus and Oral Polio Vaccines in Bangladeshi children, conducted between May 2011 and August 2018 in Dhaka, Bangladesh. The cohort consisted of 700 children enrolled within 72 hours of birth after written parental consent and were followed through twice weekly household visits and regularly scheduled clinical visits during the first 5 years of life. Details about the study design, enrollment, surveillance and biomarker development were described previously [19, 20, 22, 23]. Children were 36 months old at the time of neuro-imaging test for cognitive assessment. The study was approved by the Ethical Review Committee of the International Centre for Diarrhoeal Disease Research, Bangladesh (icddr,b), and the Institutional Review Board at Boston Childrens Hospital and the University of Virginia. This study is reported in line with the Consolidated Standards of Reporting Trials (CONSORT) Statement, and the CONSORT Checklist can be found in Additional file 2.

ERPs were measured in a subset of children at 36 months of age. After data processing and quality checking, 70 children out of 130 had valid data for the final ERP analysis. Each child was tested with a face oddball paradigm in which standard (70% of chance) and oddball (30% of chance) faces were presented in a random order. This paradigm has been widely employed to examine the neural correlates of social attention and recognition memory of faces in children [24–26]. The current study focused on one ERP component that can be elicited using this paradigm- the N290 component as the neurocognitive response. The N290 component is regarded as the precursor of the adult N170 face-sensitive component and potentially be generated by the fusiform face and occipital face areas in children [5, 27, 28]. The N290 amplitude in response to the two conditions (standard and oddball) in different electrode channels reflects the averaged synchronous brain activation of large number of neurons occurring around 290 ms following stimulus onset.

**Fig. 1:**
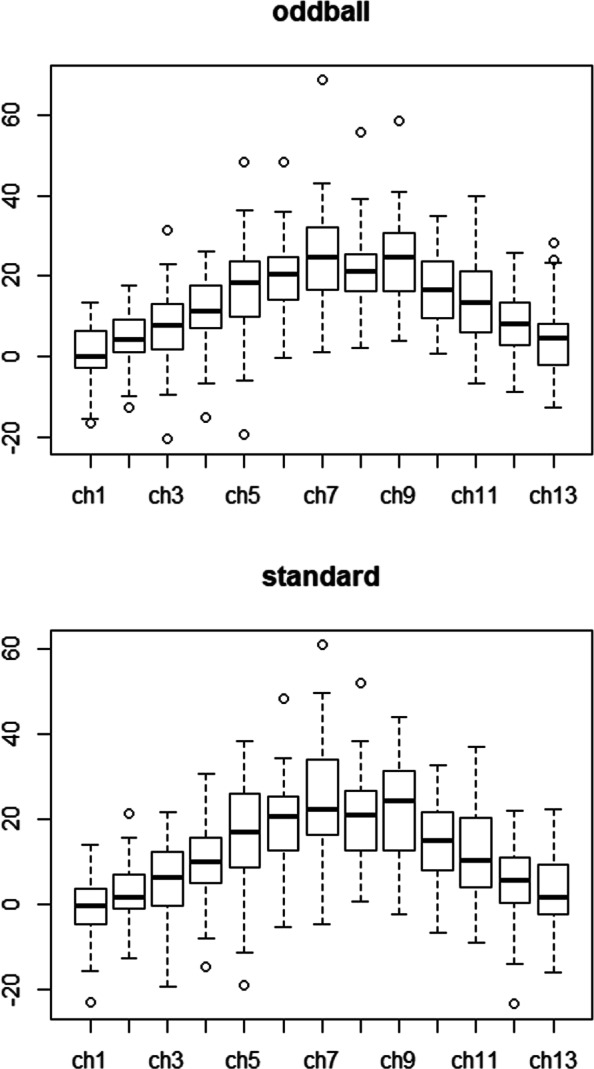
Boxplot of N290 amplitude under oddball/standard condition. X-axis represents 13 electrodes on the occipital regions of interest (see the supplemental figure for exact electrode locations). Y-axis shows the amplitude in uV

**Fig. 2:**
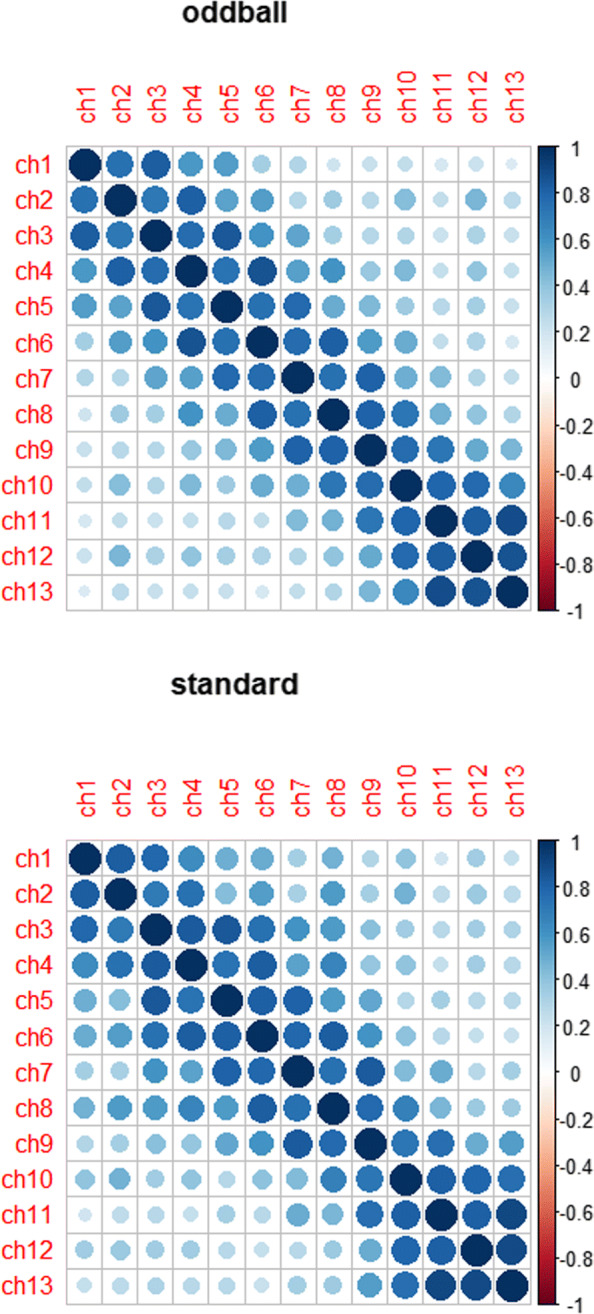
Correlation plot of N290 amplitude under oddball/standard condition

Figure 1 shows that N290 peak amplitudes are different among 13 channels (see Additional file 1 for the 13 electrode locations) under either condition, suggesting that modelling the variations among channels would capture more accurate information than simply taking average of all channels. Also, the N290 amplitudes are highly correlated among the 13 channels (Fig. 2 for the correlation plot). Furthermore, ERP response data are more structured with respect to multiple conditions by multiple channels for each subject, thus a structured correlation matrix will be needed to appropriately characterize the ERP data structure.

The clinical factors included maternal information (maternal height, weight, and education), socioeconomic status, and sanitation and environmental factors such as water source and water treatment. Biomarker data were obtained from the fecal and blood samples collected at early age of life to measure inflammation [19]. We hypothesized that only a small subset of these clinical factors and biomarkers are associated with the ERP response, thus the variable selection methods would be suitable in this investigation.

The PGEE model proposed by Wang et al. [18] for correlated data is limited in that it can only handle a simple correlation structure. While the estimator obtained by PGEE [18] is consistent with any working correlation matrix, the efficiency of the estimator can be improved when the specified correlation matrix is closer to the true matrix. To characterize the ERP correlations between conditions and among channels, we specify the within-subject correlation matrix as the Kronecker product of the channel correlation matrix and condition correlation matrix. In addition, to enable group variable selection of the categorized channel variable with 13 levels in our study, the penalty for individual variable selection in PGEE is adapted for group selection. Therefore, our method extends PGEE and prompts an integrated model for ERP responses such that we can evaluate the differences between conditions and identify informative clinical factors and biomarkers simultaneously, while accounting for the complex correlations among ERPs and allowing group variable selection.

### Proposed model for ERPs

Suppose that there are *I* subjects, each subject is placed under *J* treatments, and *K* repeated measurements are recorded under each treatment. We use *Y*_*ijk*_ to denote the *k*th repeated measures under the *j*th treatment for the *i*th subject. By vectorizing **Y**_*i*_=(*Y*_*i*11_,...,*Y*_*i*1*K*_,*Y*_*i*21_,...,*Y*_*i*2*K*_,...,*Y*_*iJ*1_,...,*Y*_*iJK*_)^*T*^, we consider group variable selection for a generalized linear model for the correlated data in **Y**_*i*_:

$$E\left(\mathbf Y_{i}\right)={\boldsymbol{\mu}_{i}},$$

$$g\left(\mu_{ijk}\right)=\mathbf X_{i}^{T} \mathbf \beta + condition_{j} + channel_{k},$$

$$Var\left(Y_{ijk}\right)=\phi v\left(\mu_{ijk}\right),$$

$$Cov\left(\mathbf Y_{i}\right) = \mathbf V_{i},$$

where **V**_*i*_ denotes the covariance structure and *ϕ* is an overdispersion parameter. Without loss of generality, we assume *ϕ*=1 in the rest of the paper.

#### Structured correlation matrix

For correlated data, a working correlation matrix needs to be pre-specified in many estimation methods, and its appropriate specification improves the estimation efficiency considerably for regression parameters. Some commonly used correlations, such as unstructured, AR1, exchangeable, etc., are often adopted in the practice. However, none of the commonly used correlations can appropriately account for the structured correlation for ERP data. Given that how ERP data were collected, it is natural to assume that ERP measurements from different conditions are correlated, and under each of treatment conditions the correlation structures among the channels are the same. Thus for the structured covariance matrix, we adopt a separate correlation for treatment condition and channel. Letting **B**_*i*_ be the covariance matrix for conditions and **Σ**_*i*_ be the covariance matrix for channels, the structured covariance matrix for each subject is the Kronecker product of the two matrices, **V**_*i*_=**B**_*i*_⊗**Σ**_*i*_.

#### Group selection for GEE

Variable selection for correlated data has been studied in Wang et al. [18], where penalized generalized estimating equations is adopted for simultaneous model estimation and variable selection, the SCAD penalty is used for individual variable selection. However, for many biomarker studies, predictors are highly correlated and/or pre-classified into different groups, and variables need to be selected in groups, as shown in Yuan and Lin [29]. Here we adopt SCAD with group selection and extend the PGEE to Group Penalized Generalized Estimating Equations (GPGEE) to select variables for the ERP data. Suppose covariates {*X*_1_,*X*_2_,...,*X*_*p*_} are classified into *d* groups

$$\left\{ X_{11},...,X_{1p_{1}} \right\},...,\left\{ X_{d1},...,X_{dp_{d}} \right\}.$$

The corresponding coefficients are $\boldsymbol {\beta }=\left (\boldsymbol {\beta ^{G}_{1}}^{T},...,\boldsymbol {\beta ^{G}_{d}}^{T}\right)$, where $\boldsymbol {\beta ^{G}_{i}}$ is the coefficient vector for group *i*. For the group variable selection, we will either select the whole group of variables or remove the whole group from the model.

We define the estimating functions as

$$U\left(\boldsymbol{\beta}\right)=S\left(\boldsymbol{\beta}\right)-n \mathbf q_{\lambda}^{G}\left(\boldsymbol{\beta}\right){sign}\left(\boldsymbol{\beta}\right),$$

where

$$S\left(\boldsymbol{\beta}\right)=\sum_{i=1}^{n}\mathbf X_{i}^{T} \mathbf A_{i}^{1/2}\left(\boldsymbol{\beta}\right)\hat{\mathbf R}^{-1}\mathbf A_{i}^{-1/2}\left(\boldsymbol{\beta}\right)\left(\mathbf Y_{i}-\mu_{i}\left(\boldsymbol{\beta}\right)\right)$$

is a vector of estimating functions defining the GEE [18], $\hat {\mathbf R}$ is the estimated working correlation matrix $\left (\mathbf V_{i} = \mathbf A_{i}^{1/2}\left (\boldsymbol {\beta }\right)\hat {\mathbf R}^{-1}\mathbf A_{i}^{-1/2}\left (\boldsymbol {\beta }\right)\right)$, and $\mathbf q_{\lambda }^{G}\left (\boldsymbol {\beta }\right)\mathbf sign\left (\boldsymbol {\beta }\right)$ denotes the component wise product with

$$sign\left(\boldsymbol{\beta}\right)=\left(sign\left(\beta_{1}\right),...,sign\left(\beta_{p}\right)\right)$$

and

$$\mathbf{q}_{\lambda}^{G}\left(\boldsymbol{\beta}\right)= \left(\boldsymbol{q}_{\lambda}\left(\left|\left|\boldsymbol{\beta}^{G}_{1}\right|\right|_{1}\right)^{T},...,\boldsymbol{q}_{\lambda}\left(\left|\left|\boldsymbol{\beta}^{G}_{d}\right|\right|_{1}\right)^{T}\right).$$

Here, $\boldsymbol {q}_{\lambda }\left (\left |\left |\boldsymbol {\beta }^{G}_{i}\right |\right |_{1}\right)= q_{\lambda }\left (\left |\left |\boldsymbol {\beta }^{G}_{i}\right |\right |_{1}\right) * \boldsymbol {1}_{p_{i}}$ denotes the group penalty vector for group *i*, and *q*_*λ*_(*θ*) is the derivative of the SCAD penalty function imposed on the *L*_1_- norm of the group vector $\boldsymbol {\beta }_{i}^{G}$.

The notation *q*_*λ*_(*θ*) is the derivative of the SCAD penalty,

$$q_{\lambda}(\theta)=\lambda \left\{I\left(\theta < \lambda \right) + \frac{\left(a\lambda-\theta \right)_{+}}{(a-1)\lambda} I\left(\theta > \lambda\right) \right\}$$

for *θ*≥0 and some *a*>2. As suggested in Fan and Li [13], we let *a*=3.7.

### Algorithm for GPGEE

Similar to the algorithm proposed in Wang et al. [18], we apply the Newton-Raphson algorithm combined with the minorization-maximization to solve the penalized estimating equations.

By the minorization-maximization algorithm, for a small *ε*>0, the penalized estimator *β*_*n*_ approximately satisfies

$$S_{nj} - nq_{\lambda_{n}}\left(\hat{\boldsymbol{\beta}}_{nj}^{G}\right) sign\left(\hat{\beta}_{nj}\right) \frac{\left|\hat{\beta}_{nj}\right|}{\epsilon + \left|\hat{\beta}_{nj}\right|} =0, j=1,..., p.$$

To solve the above equations, we apply the Newton-Raphson algorithm as follows,

$$\begin{array}{@{}rcl@{}} \boldsymbol{\beta}_{n}^{k}& = & \boldsymbol{\beta}_{n}^{k-1} + \left[\boldsymbol{H}_{n}\left(\boldsymbol{\beta}_{n}^{k-1}\right) + n\boldsymbol{E}_{n}\left(\boldsymbol{\beta}_{n}^{k-1}\right)\right]^{-1}\\ && \times \left[\boldsymbol{S}_{n}\left(\boldsymbol{\beta}_{n}^{k-1}\right) - n\boldsymbol{E}_{n}\left(\boldsymbol{\beta}_{n}^{k-1}\right) \boldsymbol{\beta}_{n}^{k-1}\right], \end{array} $$

where

$$\boldsymbol{H}_{n}\left(\boldsymbol{\beta}_{n}^{k-1}\right) = \sum_{i=1}^{n} \mathbf X_{i}^{T} \mathbf A_{i}^{1/2}\left(\boldsymbol{\beta}_{n}^{k-1}\right)\mathbf R^{-1}\mathbf A_{i}^{1/2}\left(\boldsymbol{\beta}_{n}^{k-1}\right) \mathbf X_{i},$$

$$\boldsymbol{E}_{n}\left(\boldsymbol{\beta}_{n}^{k-1}\right) = diag \left\{ \frac{q_{\lambda_{n}}\left(\boldsymbol{\hat{\beta}_{n1}^{G}}\right)}{\epsilon + \left|\hat{\beta}_{n1}\right|},..., \frac{q_{\lambda_{n}}\left(\boldsymbol{\hat{\beta}_{np}^{G}}\right)}{\epsilon + \left|\hat{\beta}_{np}\right|} \right\}.$$

In practice, we set *ε*=10^−6^ and take $\hat {\boldsymbol {\beta }}$, the GEE estimator with independence working correlation matrix, as the initial value of *β*. The stopping criterion for the iterative algorithm is $\sum _{j=1}^{p}\left |\hat {\boldsymbol {\beta }}_{j}^{k+1}-\hat {\boldsymbol {\beta }}_{j}^{k}\right | < 10^{-5}$. In our study, we use Bayesian information criterion (BIC) developed for correlated data [30] for selecting the tuning parameter *λ*.

## Simulations

In this section, we illustrate the numerical strength of our developed method by comparing it with existing methods through a simulation study. In our simulation study, the sample sizes are set at 50 and 100. There are 20 correlated measurements and 40 covariates for each subject. The correlated normal responses are generated from the model

$$Y_{ij}=\boldsymbol{X}^{T}_{ij} \boldsymbol{\beta} + \epsilon_{ij},$$

where $\boldsymbol {X}^{T}_{ij}=\left (x_{ij,1},..., x_{ij, 40}\right)^{T}$ is a vector of 40 covariates for *i*=1,..,50 and *j*=1,...,20, and

$${} \boldsymbol{\beta}\,=\,\left(2, 1, 1, 1, 1, 3, 3, 3, 3, 0, 0, 0, 0, 0, 0.1, 0.1, 1, 1, 1, 0,..., 0\right)^{T}$$

containing 8 groups with every 5 covariates in each group. For the covariates, we generate *x*_*ij*,1_ from Bernoulli(0.5) distribution and the rest from the multivariate normal distribution with mean 0 and an AR1 covariance matrix with marginal variance 1 and auto-correlation coefficient 0.5. The covariance matrix of random errors for each subject is **V**_*i*_=**B**_*i*_⊗**Σ**_*i*_, where **B**_*i*_ is a 2-by-2 identity matrix and **Σ**_*i*_ is a 10-by-10 AR1 matrix with marginal variance 10 and auto-correlation coefficient 0.9. We compare our GPGEE model with PGEE model to illustrate the importance of incorporating group penalty and using structured correlations. Five models are evaluated for comparison in the simulations: original PGEE with AR1 working correlation (Model 1), a modified PGEE incorporated with our structured correlation (Model 2), our GPGEE with AR1 working correlation (which is unstructured correlation, Model 3), our GPGEE with structured correlation but with misspecified working correlation (Model 4) and our proposed model with both group penalty and correct structured correlation (Model 5). We assume that the true group memberships of the covariates to impose the group-SCAD penalty are known, and divide the covariates into 8 groups. For the structured correlation that is correctly specified, we use AR1 as working correlation structure for **Σ**_*i*_ and assume **B**_*i*_ to be unstructured. For the misspecified structured correlation, we use CS (compound symmetry) as working correlation structure for **Σ**_*i*_ instead. The selection results are defined as exact-selection when the selected model is the true model, under-selection when at least one true covariate is not selected, and all other cases are defined as over-selection. We also report the mean squared error (MSE) which is defined as the average of $\left |\left | \hat {\boldsymbol {\beta }} - \boldsymbol {\beta } \right |\right |^{2}_{2}$ and corresponding standard error (SE) defined as the standard deviation of $\left |\left | \hat {\boldsymbol {\beta }} - \boldsymbol {\beta } \right |\right |^{2}_{2}$ from the simulated datasets.

We conduct the simulation by generating 200 datasets for each sample size, and summarize the percentages of over-selection, under-selection, exact-selection, and MSEs (SE) in Table 1, top panel for sample size 50. Our GPGEE model (Model 5) has the smallest MSE and SE among the 5 models, and selects the true model for 94.5% of the simulated datasets, compared to the existing PGEE model (Model 1) with only 2.5% exact-selection and much higher MSE. The results further show that without the pre-specified structured correlation (Model 3), the model selection is less accurate, and it is more likely to have higher under-selection and larger MSE (SE), which suggests it is crucial to incorporate the structured correlation when the data is multi-level by nature. The results also show the importance of adopting group penalty, especially to deal with under-selection problem when there are covariates with smaller coefficients. In addition, if we capture the multi-level correlation structure correctly but mis-specify the true correlation for one layer (Model 4), the method can still be helpful to select the true model (96% selection rate) though the MSE is larger due to the mis-specification. Thus, as long as we specify this multi-level correlation structure, our method is relatively robust for variable selection against misspecification of the working correlation structure. When the sample size is increased 100, the comparsion results remain to be similar, as shown in Table 1, bottom panel.

**Table 1:** Comparison of model selection performance. O for over-selection, U for under-selection and Exact for exact-selection

Model	Working correlation matrix	O	U	Exact	MSE (SE)
Sample size = 50					
Model 1: PGEE	AR1	15.5%	82.0%	2.5%	0.47 (0.35)
Model 2: PGEE	unstructured ⊗ AR1	19.0%	79.0%	2.0%	0.45 (0.33)
Model 3: GPGEE	AR1	1.0%	5.5%	93.5%	0.49 (0.25)
Model 4: GPGEE	unstructured ⊗ CS	0.0%	4.0%	96.0%	0.37 (0.18)
Model 5: GPGEE	unstructured ⊗ AR1	2.0%	3.5%	94.5%	0.24 (0.13)
Sample size = 100					
Model 1: PGEE	AR1	11.0%	75.0%	14.0%	0.25 (0.16)
Model 2: PGEE	unstructured ⊗ AR1	33.5%	53.0%	13.5%	0.16 (0.08)
Model 3: GPGEE	AR1	1.5%	2.0%	96.5%	0.18 (0.11)
Model 4: GPGEE	unstructured ⊗ CS	1.5%	0.5%	98.0%	0.21 (0.12)
Model 5: GPGEE	unstructured ⊗ AR1	1.0%	0.5%	98.5%	0.13 (0.07)

## Results

In the PROVIDE cohort, there were 47 clinical factors and early-stage biomarkers available for the analysis, including children’s enteric and systemic inflammatory biomarkers, nutritional measures, maternal health and socioeconomic status (SES), and sanitation conditions [19]. Of them, 14 biomarkers are categorical measures, and the rest are continuous variables. The CRP index is a cumulative number of times that children experienced elevated CRP level over the first two years (i.e., being on the top 50% at 6, 18, 40, 53 and 104 weeks), thus measuring the sustained inflammation burden. For 70 children with ERP measurements, the descriptive statistics of these clinical factors and biomarkers are summarized in Table 2.

**Table 2:** Descriptive summary of risk factors and biomarkers in ERP Study (N=70)

Category	Risk factor/Biomarker	Child age (week)	Mean ± SD or percentage
Enteric inflammation	Myeloperoxidase (MPO)	12	10057.57 ±9189.827
	Calprotectin	12	933.41 ±679.94
	Neopterin	12	2468.82 ±1644.28
	Alpha-1 anti-trypsin (ALA)	12	0.88 ±0.62
	Mannitol in urine	12	0.015 ±0.017
		24	0.019 ±0.018
	Lithostathine-1-beta (Reg1B)	6	59.50 ±84.59
		12	59.89 ±72.70
	Days of diarrhea	18	6.39 ±8.31
Systemic inflammation	Ferritin	6	175.99 ±104.08
		18	28.60 ±25.32
	C reactive protein (CRP) index	6, 18, 40, 53, 104	2.51 ±1.19
	Soluble CD14	6	1736.29 ±568.87
		18	2284.00 ±787.66
	Endocab lipopolysaccharide (LPS)	6	37.11 ±5.57
		18	29.75 ±71.70
	Log scale of activin	6	6.49 ±1.13
	Interleukin 1 beta (IL1b)	18	37.1% (top 50%)
	Interleukin 4 (IL4)	18	44.3%
	Interleukin 5 (IL5)	18	38.6% (top 50%)
	Interleukin 6 (IL6)	18	55.7% (top 50%)
	Interleukin 7 (IL7)	18	75.7% (top 50%)
	Interleukin 10 (IL10)	18	64.3% (top 50%)
	Macrophage inflammatory protein 1 Beta (MIP1b)	18	38.6% (top 50%)
	Tumor necrosis factor alpha (TNFa)	18	41.4% (top 50%)
Nutritional	Vitamin D	6	28.10 ±14.26
		18	52.85 ±22.50
	Zinc	6	690.30 ±105.64
		18	768.41 ±136.78
	Retinol binding protein (RBP)	6	28846.90 ±11315.83
		18	36762.30 ±15422.36
	Height for age z score (HAZ)	Birth	-0.95 ±0.79
	Weight for age z score (WAZ)	Birth	-1.28 ±0.83
	Weight for height z score (WHZ)	Birth	-1.22 ±0.96
	Days of exclusive breast milk feeding	18	102.81 ±40.06
Maternal health, SES	Monthly household expenditure	Enrollment	12112.86 ±6761.06
	Monthly household income	Enrollment	13505.71 ±8680.46
	Mother height (cm)	Enrollment	149.82 ±5.74
	Mother weight (kg)	Enrollment	49.33 ±10.91
Sanitation	Access to treated water	Enrollment	58.6%
	Access to toilet with a septic tank	Enrollment	67.1%
	Access to private toilet not shared with neighbors	Enrollment	10.0%
	Covered drain near home	Enrollment	65.7%

As described earlier, in the ERP study, the children were shown with the face pictures, 70% of time for the same face (standard condition) and 30% of time with new different faces (oddball condition) over 150 trials. Brain activities were recorded for all electrodes during the observation of each picture, and ERP components were derived from multiple trials to measure the electrical activity of the brain immediately in response to a direct stimulus event [6]. In this clinical application, the mean peak amplitude of N290 component was used as a clinical example, which measures the brain response with face processing around 290 ms, obtained under each treatment condition from 13 electrodes placed on different locations of occipital region. The N290 amplitude reflects the synchronous activation of large number of neurons, and large amplitude is generally deemed to have greater underlying neuronal activity. It is hypothesized that the N290 amplitude response originates in areas of the brain dedicated to face processing, such as the occipital face areas and the inferior temporal cortex (such as the fusiform). In addition to evaluate the difference in N290 amplitude for neural activity of face processing between the two conditions, we aimed to study the association of biomarkers in infancy with the ERP response in early childhood. Ultimately, we hope to gain insights on how infant’s health and nutrition markers affects the development of the brain.

For each child, there are 26 N290 amplitude responses corresponding to 13 channels under 2 conditions. Those 26 ERP responses are highly correlated with multilevel correlation structure due to the nature of this experiment, that is, N290 measurements are not only correlated across channels, but also vary under different treatment conditions. As shown in Fig. 2, the N290 measurements among 13 channels (aligned by their locations on the brain) are highly correlated, and the correlations appear to be autoregressive in that channels closer to each other in the brain yield higher correlations than that further apart. Also, the correlation patterns appear to be different between oddball and standard conditions. In addition, N290 measurements vary considerably across the 13 channels and across conditions as depicted in Fig. 1. For the special data features, our proposed GPGEE model described in “Methods” section can properly evaluate the relationship between biomarkers and N290 response while accounting for the hierarchical correlation structures and variations across channels/conditions. To apply our proposed model, the correlation matrix between conditions for the same channel was assumed to be unstructured, and that among channels for the same condition to be autoregressive with order 1 (AR1). The group penalty was applied to electrode or channel which is a multi-level categorical covariate with 13 levels. By using 12 dummy variables and grouping them together, we are able to conduct variable selection for this covariate. The N290 responses were assumed to be normally distributed with identity link. For biomarkers and clinical predictors, prescreening was performed based on their correlations, and representative predictors were selected for those with corrections >0.7. Thus 6 biomarkers were removed, including IL-4 at week18, IL-6 at week 18, TNFa at week 18, WAZ at birth, WHZ at birth and monthly household income.

The results of variable selection with our proposed GPGEE for N290 response were presented in Table 3. A total of 10 biomarkers were selected using BIC after adjusting for condition and channel differences. Among those selected biomarkers, IL-10, RBP, Zinc, Calprotectin, Neopterin and water treatment were positively associated with N290 amplitude, while IL-5, MIP1b, MPO and maternal height have negative effects on the N290. These results provide some supporting evidence that children’s health conditions in early childhood indeed are associated with brain development at 3 years of age. While N290 amplitude measures the strength of the signal of brain activity for brain connectivity, some researchers have also focused on studying the change of N290 amplitude between conditions. The differences in N290 between oddball and standard conditions reflects how the brain behaves differently when seeing a new face vs. a familiar face, and therefore measures the child’s ability to discriminate between a novel and a familiar face. In particular, A differential response in these ERP components between the two experimental conditions indicates the detection or discrimination of the infrequent from the frequent faces by the brain and reflects some aspect of memory updating and the efficiency of stimulus processing [26, 31, 32].

**Table 3:** Risk factors and biomarkers selected for N290 amplitude

Risk factor/Biomarker	Effect
IL-5 at week 18	-
IL-10 at week 18	+
MIP1b at week 18	-
RBP at week 6	+
Zinc at week 18	+
MPO at week 12	-
Calprotectin at week 12	+
Neopterin at week 12	+
Mother height	-
Water treatment	+

When considering the difference in N290 as the outcome variable, the analysis would be performed similarly under our GPGEE framework, where the correction structure is reduced at channel level only. For the difference in N290 response, 13 biomarkers were selected (Table 4), of which 8 biomarkers have positive effects and 5 have negative effects on the N290 difference. Obviously, RBP at week 6, Zinc, mother height, and water treatment were associated with both N290 amplitude and the difference in N290, while some biomarkers (Days of diarrhea in the first 18 weeks, RBP at week 18, Mannitol in urines at week 24, LPS at week 18, CRP index, Monthly household expenditure, Mother weight, Reg1B at week 6, Gender) were only informative to the difference of N290 between the conditions, indicating that these biomarkers contributing to a stronger overall brain EEG signal don’t necessarily contribute to a better EEG power, the ability to identify new faces.

**Table 4:** Risk factors and biomarkers selected for N290 difference

Risk factor/Biomarker	Effect
Days of diarrhea in the first 18 weeks	+
RBP at week 6	+
RBP at week 18	-
Zinc at week 18	+
Mannitol in urines at week 24	+
LPS at week 18	-
CRP index	+
Monthly household expenditure	-
Mother weight	+
Mother height	+
Reg1B at week 6	+
Gender	-
Water treatment	-

## Discussion

The primary objective of our clinical study was to identify biomarkers in early childhood that could affect children’s brain development measured by ERP data at 3 years of age. To our best knowledge, no previous study has analyzed ERP data under correlated hierarchical data framework where the correlation structure among both channels and conditions are accounted for. Many available statistical methods couldn’t be directly applied here because of the nature of ERP’s correlation structure. Further, group penalty needs to be incorporated in variable selection for ERP data so the clustered clinical risk factors and biomarkers can be selected together. Therefore our proposed group penalized GEE estimator with structured correlation matrix for ERP data can properly model the complex ERP response and simultaneously identify informative biomarkers associated with ERP amplitude and ERP difference, respectively. Our proposed method outperforms the existing modeling approaches in the simulation study. Further, our work would be one of the pioneering efforts in ERP research to test the condition difference in ERPs and, simultaneously, to identify important covariates associated with ERPs.

In our study, N290 measure was analyzed in the clinical application, but the developed method can be applied to any other ERP measurements with tasks focusing on different brain functions. Our clinical findings were limited by the small sample size, missing data in biomarkers, and time lag between collection of biomarkers and ERP measurement. Nevertheless, our proposed method emphasizes on the correlation structure among channels based on their physical locations on the brain, thus improves the model estimation efficiency for ERP data analysis. For future work, if data is normally distributed with identity link function, our proposed method can be extended further with choices of penalty, such as elastic net, and computing algorithms, such as Fast Iterative Shrinkage-Thresholding Algorithm [33] or Alternating Direction Method of Multipliers [34], which would improve the computational time for larger datasets. In addition, although the ERP responses were considered as the continuous outcomes, our model is also applicable to other types of response such as categorical or count response. In addition, the systemic and enteric inflammation biomarkers identified in this study for their association with ERPs are similar and consistent with the previous findings in the cognitive development research [35].

## Conclusions

Using the proposed group penalized GEE, we modeled the complex ERP data with structured correlation and identified informative early-stage biomarkers associated with such brain connectivity. Our findings are clinically important in understanding early childhood neurocognitive development in low-income countries. Particularly, the selected early-stage biomarkers offer a potential explanation for the adversity of brain connectivity, which will facilitate early identification of infants at risk and potential pathways for effective intervention in the malnourished children. Our proposed method is not only applicable to the ERP studies but also to other biomedical studies for biomarker selection with highly correlated responses.

## Supplementary information

**Additional file 1** Supplemental figure and table. Electrode locations, corresponding electrode numbers and channel numbers used in the paper.

**Additional file 2** CONSORT checklist for the related clinical study.

## Data Availability

The datasets supporting the conclusions of this article are available at icddr,b, and at Boston Children’s Hospital and University of Virginia. According to the data protection regulation and informed consent form, the authors are not permitted to deposit the individual participant data elsewhere.

## Abbreviations

ERP: Event-Related Potentials
GEE: Generalized Estimating Equation
PGEE: Penalized Generalized Estimating Equation
GPGEE: Group Penalized Generalized Estimating Equation
EEG: Electroencephalogram
LASSO: Least Absolute Shrinkage and Selection Operator
SCAD: Smoothly Clipped Absolute Deviation
PROVIDE: Performance of Rotavirus and Oral Polio Vaccines in Developing Countries
BIC: Bayesian Information Criterion
SES: Socioeconomic Status
AR1: Autoregressive with Order 1
MPO: Myeloperoxidase
ALA: Alpha-1 Anti-trypsin
Reg1B: Lithostathine-1-Beta
CRP: C Reactive Protein
LPS: Endocab Lipopolysaccharide
L1b: Interleukin 1 beta
IL4: Interleukin 4
IL5: Interleukin 5
IL6: Interleukin 6
IL7: Interleukin 7
IL10: Interleukin 10
MIP1b: Macrophage Infammatory Protein 1 Beta
TNFa: Tumor Necrosis Factor alpha
RBP: Retinol Binding Protein
HAZ: Height for Age Z score
WAZ: Weight for Age Z score
WHZ: Weight for Height Z score
