# Supplementary material for: Group penalized generalized estimating equation for correlated event-related potentials and biomarker selection
Source: BMC Med Res Methodol. 2020 Aug 31;20:221. doi: 10.1186/s12874-020-01103-x (PMC7457526; doi:10.1186/s12874-020-01103-x)
Supplement: Supplementary file 1 — Additional file 1 Supplemental figure and table. Electrode locations, corresponding electrode numbers and channel numbers used in the paper. [file 12874_2020_1103_MOESM1_ESM.docx]

**Supplemental document**

**­­­**


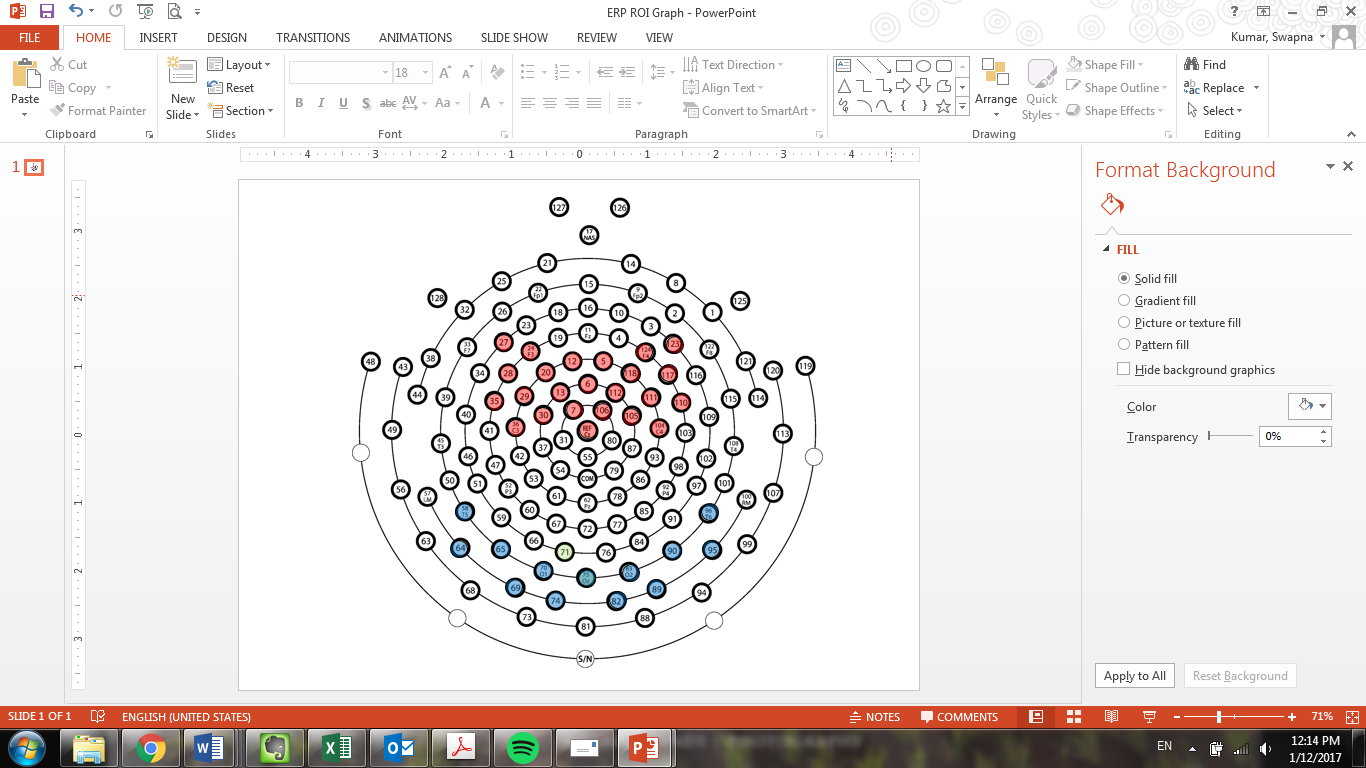
­­

Supplemental figure 1. Electrodes locations and numbers.

| Channel number | Corresponding electrode number |
| --- | --- |
| 1 | 58 |
| 2 | 64 |
| 3 | 65 |
| 4 | 69 |
| 5 | 70 |
| 6 | 74 |
| 7 | 75 |
| 8 | 82 |
| 9 | 83 |
| 10 | 89 |
| 11 | 90 |
| 12 | 95 |
| 13 | 96 |

Supplemental table 1. Channel numbers and corresponding electrode numbers.
